# Enhanced interlayer trapping of Pb(II) ions within kaolinite layers: intercalation, characterization, and sorption studies

**DOI:** 10.1007/s11356-019-06845-w

**Published:** 2019-11-23

**Authors:** Ali Maged, Ismael Sayed Ismael, Sherif Kharbish, Binoy Sarkar, Sirpa Peräniemi, Amit Bhatnagar

**Affiliations:** 1 Department of Environmental and Biological Sciences, University of Eastern Finland, P.O. Box 1627, FI-70211 Kuopio, Finland; 2 Geology Department, Faculty of Science, Suez University, P.O. Box 43518, El Salam City, Suez Governorate Egypt; 3 Department of Animal and Plant Sciences, The University of Sheffield, Western Bank, Sheffield, S10 2TN UK; 4 Lancaster Environment Centre, Lancaster University, Lancaster, LA1 4YQ UK; 5 School of Pharmacy, University of Eastern Finland, FI-70211 Kuopio, Finland

**Keywords:** Kaolinite, Intercalated kaolinite, Adsorption, Lead removal, Water treatment

## Abstract

Lead (Pb(II)) pollution in water poses a serious threat to human health in many parts of the world. In the past decades, research has been aimed at developing efficient and cost-effective methods to address the problem. In this study, dimethyl sulfoxide (DMSO) and potassium acetate (K-Ac) intercalated kaolinite complexes were synthesized and subsequently utilized for Pb(II) removal from water. The intercalation of kaolinite with DMSO was found to be useful for expanding the interlayer space of the clay mineral from 0.72 to 1.12 nm. Kaolinite intercalation with K-Ac (KDK) increased the interlayer space from 1.12 to 1.43 nm. The surface area of KDK was found to be more than threefold higher as compared to natural kaolinite (NK). Batch experimental results revealed that the maximum Pb(II) uptake capacity of KDK was 46.45 mg g^−1^ which was higher than the capacity of NK (15.52 mg g^−1^). Reusability studies showed that KDK could be reused for 5 cycles without substantially losing its adsorption capacity. Furthermore, fixed-bed column tests confirmed the suitability of KDK in continuous mode for Pb(II) removal. Successful application of intercalated kaolinite for Pb(II) adsorption in batch and column modes suggests its application in water treatment (especially removal of divalent metals).

**Electronic supplementary material:**

The online version of this article (10.1007/s11356-019-06845-w) contains supplementary material, which is available to authorized users.

## Introduction

Clay minerals are one of the natural and plentiful materials on the earth. Clay has a long history of utilization in the industrial, agricultural, health, and environmental sectors. Clay minerals are hydrous aluminosilicates (silica tetrahedral sheets and alumina octahedral sheets) and widely defined as minerals that are composed of the colloidal fraction of rocks, soils, and sediments. Clay minerals can be grouped under different types such as 1:1 type (kaolinite and halloysite), 2:1 type (smectite and vermiculite), and 2:1:1 type (chlorite). Moreover, modified clay minerals are widely used as sorbents for various traditional and emerging contaminants, as well as in cosmetics, paints, and thixotropic and fluidic agents (de Paiva et al. 2008). Among other clay minerals, kaolinite is an excellent eco-friendly material and has been used in various industries for a variety of applications.

Kaolinite (Al_2_Si_2_O_5_(OH)_4_) is a 1:1 type layered structure clay mineral and it is one of the inexpensive and abundant clay minerals (Zhang et al. 2018). Due to the low specific surface area and cation exchange capacity (CEC) (3 to 15 meq/100 g), as compared to other clay minerals, modification is required to raise the adsorption efficacy of kaolinite. Modification of kaolinite can be done by intercalating, pillaring, or organic and inorganic grafting of molecules on its surface, which improve the CEC of kaolinite products (Guerra et al. 2008). The intercalation of clay mineral can be defined as the insertion of a new molecule(s) in the interlayer of a clay mineral while keeping the layered structure preserved (Matusik and Kłapyta 2013).

Kaolinite presents two chemically different interlayer structures: alumina (Al^3+^) octahedral sheet covered with hydroxyl groups and silica (Si^4+^) tetrahedral sheet covered with basal oxygens. The neighboring kaolinite layers form hydrogen bonds, dipole–dipole interactions, and van der Waals forces between the hydroxyl groups of the octahedral sheet and the basal oxygens on the tetrahedral sheet of the adjacent layer to hold them together (Zhang et al. 2014). This is due to the presence of hydrogen-bonding between the surfaces. Kaolinite was previously classified as a nonexpandable clay mineral; however, there is evidence that a few polar organic compounds, such as dimethylformamide (Churchman et al. 1984), urea (Makó et al. 2009), acetamide, pyridine N-oxide, methanol, octadecyl amine, dimethyl sulfoxide (Costanzo 1986), and potassium acetate (Frost et al. 2000), can be directly intercalated in its interlayer space (Li et al. 2008). Thus, the modification of kaolinite by intercalation can improve the physical and chemical properties of the clay mineral, which can be used as an adsorbent for different metal ions such as Pb(II) ions from contaminated water.

Pb(II) is a widely used in industrial applications, and it is considered as the third-most common toxic element in the list of heavy metals (Yin et al. 2018). The World Health Organization (WHO) listed Pb(II) as one of the 10 chemicals that have significant public health concerns (WHO 2011a). Pb(II) enters into the aquatic environment through natural and anthropogenic sources and can accumulate in the food chain (EFSA 2010; Mager 2011). Exposure to the high concentration of Pb(II) could compromise the central nervous system and kidneys (Anitha et al. 2015a); impair immune, digestive, and reproductive systems in humans; and cause severe health risks, especially to children’s health (Kumar et al. 2011; Li et al. 2014). The WHO has set 0.01 mg L^−1^ as the acceptable limit for Pb(II) in potable water (WHO 2011b). In industrial wastewater, Pb(II) concentration might be as high as 20–400 mg L^−1^ (Wei et al. 2016).

Adsorption is considered as one of the most successful and effective methods for Pb(II) removal from contaminated water, owing to the low cost and ease of operation and regeneration possibility of the spent adsorbents (Senthil Kumar et al. 2013; Sdiri et al. 2016; Gunasundari and Kumar 2017). Activated carbon is commonly utilized as an adsorbent in wastewater treatment (Neeraj et al. 2016; Saravanan et al. 2018). However, activated carbon is expensive and needs chelating to increase the metal ion’s adsorption capacity due to its hydrophobic surface (Kiruba et al. 2014). Such disadvantages increase the financial cost of water treatment. Seeking a highly efficient, cost-effective, and abundantly available adsorbent to remove Pb(II) from wastewater has become a subject of considerable interest. One of the best candidate adsorbents for this purpose can be natural and modified clay minerals (Rusmin et al. 2015; Perelomov et al. 2016; Shaban et al. 2017, 2018b).

This research aimed to modify an Egyptian kaolinite clay mineral in order to increase its potential for Pb(II) removal from aqueous media. A thermal modification method and intercalation with DMSO and K-Ac were applied. The lead-trapping performance of the modified kaolinite was studied. The effects of various parameters were tested in batch mode studies. The adsorption rates were determined quantitatively and assessed by the pseudo-first-order (PFO), pseudo-second-order (PSO), and intraparticle diffusion (IPD) kinetic models. In addition, several well-known isotherm models, namely Langmuir, Freundlich, Sips, and Redlich–Peterson isotherm models, were applied to fit the investigated data. Finally, Pb(II) removal using modified kaolinite was validated in fixed-bed columns to demonstrate its feasibility for real-scale water treatment.

## Materials and methods

### Clay mineral

The kaolinite sample, selected for this study, was collected from Abu Zenima, South Sinai Governorate, Egypt. The CEC of this sample was 11 meq/100 g. The chemical composition of the raw kaolinite in mass % basis was as follows: SiO_2_, 54.64; Al_2_O_3_, 28.76; TiO_2_, 1.73; Fe_2_O_3_, 2.55; MgO, 0.35; CaO, 0.09; K_2_O, 0.19; Na_2_O, 0.21; and P_2_O_5_, 0.05; and loss on ignition was 11.36.

### Reagents

The Pb(II) solution was prepared using Pb(NO_3_)_2_∙4H_2_O (purity > 99.999%). Sodium hydroxide (NaOH), dimethyl sulfoxide (DMSO), potassium acetate (K-Ac), hydrochloric acid (HCl), sodium acetate (Na-Ac), and hydrogen peroxide (H_2_O_2_; 30% w/w) were of analytical grade (purities of at least 98%) and purchased from Sigma-Aldrich Oy, Finland.

### Adsorbent preparation

#### Kaolinite pretreatment

The kaolinite was washed twice with Milli-Q water (18.2 MΩ cm) (MQW) and dried. Thereafter, the sample was ground and sieved (less than 200 μm). To remove the impurities (carbonates and organic matter), 30 g of the kaolinite was treated with 500 mL of a buffer solution (composed of Na-Ac (0.1 N) and acetic acid solution) followed by the gradual addition of 100 mL H_2_O_2_. Next, the slurry was stirred with a magnetic stirrer for 10 h at 70 ± 1 °C followed by 24 h at ambient temperature. Thereafter, the suspension was centrifuged (model 5430, Eppendorf AG, Germany) at 4000 rpm for 15 min and filtered. The residue was then washed three times with 0.01 N NaCl solution and placed overnight at 105 ± 1 °C in an air oven. Thereafter, the kaolinite (labeled “NK”) was ground, sieved using 100 μm sieve, and stored in a glass vial until further use.

#### Kaolinite intercalation method

Intercalation of kaolinite (NK) with DMSO (NK-DMSO) was done as follows: (a) 25 g of NK was calcined at 400 ± 1 °C for 4 h. The calcination was performed in order to increase the kaolinite surface area before the intercalation, which also increases the intercalation possibilities; (b) 20 g of the thermal pre-activated NK was added to 200 mL of DMSO under continuous stirring at 20 rpm for 72 h at 60 ± 1 °C; (c) NK-DMSO was rinsed several times with solution of MQW and methanol (10%), centrifuged, and dried in an air oven (60 ± 1 °C) for 72 h to eliminate excess DMSO.

The intercalation of NK-DMSO with K-Ac was conducted in the following steps: (a) 15 g of NK-DMSO was added to 50 mL of K-Ac solution at 30% mass percentage concentration and stirred (48 h) at 20 rpm at ambient temperature; (b) the solution was filtered, washed, and dried (70 ± 1 °C) in an air oven for 24 h to evaporate excess water. Finally, the modified kaolinite was ground, sieved using a 100-μm sieve, and stored in a glass vial (labeled “KDK”) until further use in adsorption experiments.

### Physicochemical analysis

The NK, NK-DMSO, and KDK samples were characterized by several characterization techniques to get an insight into the physicochemical properties of the kaolinite samples. The detailed information about the methodology and instrumentation are given in the Supplementary material.

### Batch adsorption studies

Batch experiments were conducted with NK and KDK samples to determine their potential toward Pb(II) removal from water. Pb(II) solution (300 mg L^−1^) was prepared and diluted further as needed. All batch experiments were done at room temperature (25 ± 1 °C) in 100 mL capped glass bottles (Schott Duran, Germany) containing 50 mL of metal ion solutions agitated at 200 rpm on an orbital shaker. The adsorption parameters, such as solution pH (2–9), adsorbent dose (0.1–8 g L^−1^), contact time (0–300 min), ionic strength (0.1–0.5 M NaCl), and initial adsorbate concentration (5–250 mg L^−1^), were conducted in order to optimize the condition. All the experiments were done in duplicate. To study the effect of individual parameters, other parameters were kept constant while varying that particular parameter. After equilibration, 10 mL of the solution was withdrawn and filtered. Thereafter, the Pb(II) concentration in the filtrate was determined using atomic absorption spectroscopy (AAS) (Analytik Jena ZEEnit 700, Germany).

The Pb(II) adsorption capacity and removal percentage by the clay adsorbents were calculated using the following Eqs. (1)–(2):

1 $$ {q}_e=\frac{C_i-{C}_e}{w}\times V $$

2 $$ Removal\%=\frac{C_i-{C}_e}{C_i}\times 100 $$

### Reusability studies

The regeneration experiments were performed with NK and KDK under the optimum condition based on the optimization studies. The adsorbents were added to a solution containing 40 mg L^−1^ of Pb(II) and shaken for 3 h to achieve equilibrium. Then, the separated adsorbents were washed and dried at 60 °C for 4 h. A known volume of HCl (0.1 M) was selected as the regeneration agent and agitated for 3 h. Five cycles of adsorption/desorption were carried out accordingly. Finally, the Pb(II) concentration in the solution after each cycle was analyzed using AAS.

### Theoretical models

#### Adsorption kinetic modeling

Kinetic studies of metal removal by an adsorbent are important for evaluating the rate and mechanism of adsorption. In this study, PFO (Eq. (3)) (Lagergren 1898), PSO (Eq. (4)) (Ho and McKay 1999), and IPD (Eq. (5)) (Weber and Morris 1963) were utilized to get insight into the adsorption mechanism. The experimental data were fitted to these models using the nonlinear regression in MATLAB software (R2018b).

3 $$ {q}_t={q}_e\left(1-{e}^{-{k}_1t}\right) $$

4 $$ {q}_t=\frac{k_2{q}_e^2t}{1+{k}_2{q}_et} $$

5 $$ {q}_t={k}_{id}{t}^{1/2}+C $$

#### Adsorption isotherm modeling

The adsorption isotherm represents adsorbent and adsorbate interaction at variable initial concentrations. Four models were selected to define the Pb(II) adsorption onto NK and KDK: Langmuir (Eq. (6)) (Langmuir 1918), Freundlich (Eq. (7)) (Freundlich 1924), Sips (Eq. (8)) (Sips 1948), and Redlich–Peterson (Eq. (9)) (Redlich and Peterson 1959). MATLAB (R2018b) was used to calculate the model’s parameters by nonlinear regression modeling with the experimental data.

6 $$ {q}_e=\frac{q_m{K}_L{C}_e}{1+{K}_L{C}_e} $$

7 $$ {q}_e={K}_F{C}_e^{\raisebox{1ex}{$1$}\!\left/ \!\raisebox{-1ex}{$n$}\right.} $$

8 $$ {q}_e=\frac{K_s{C}_e^{\beta s}\ }{1+{a}_s{C}_e^{\beta s}} $$

9 $$ {q}_e=\frac{K_R{C}_e\ }{1+{\alpha}_R{C}_e^g} $$

The *R*^2^ and RMSE values were used to designate the best fit of the experimental data with the models. The lower RMSE value defines the data more precisely for this model. The RMSE can be calculated using Eq. (10) (Hafshejani et al. 2017).

10 $$ RMSE=\sqrt{\frac{\sum_1^N{\left(\ {q}_{exp}-{q}_{model}\ \right)}^2}{N}} $$

where *q*_exp_ and *q*_model_ are the experimentally measured value and model prediction for Pb(II) adsorption, respectively.

### Fixed-bed column adsorption studies

#### Column preparation

The fixed-bed column studies were conducted with KDK using a laboratory-scale glass column (column length of 130 mm and an internal diameter of 5 mm) assembly. The adsorbent was sandwiched in between glass wool layers to prevent the adsorbent loss, and the column was closed to ensure good distribution of the liquid phase. The column was packed with 100 and 200 mg of KDK to obtain an adsorbent bed height approximately equivalent to 5 and 10 mm, respectively. Two different flow rates (1.5 and 3 mL min^−1^) were pumped to elucidate the effect of influent flow. Similarly, two different solutions having Pb(II) concentrations of 10 and 20 mg L^−1^ were utilized to explicate the effect of influent concentration (*C*_o_). In the process, the samples were collected at different time intervals to determine the residual Pb(II) concentration in the effluent solutions. Finally, the column operation was stopped when there was no further Pb(II) adsorption (the influent and effluent concentrations appeared similar).

#### Column data analysis

The effluent samples were analyzed for Pb(II) concentration using AAS. The performance of the fixed-bed column was represented by the breakthrough curve. The breakthrough point (*t*_b_) shows when the effluent concentration *C*_*t*_/*C*_o_ > 0.05 of the influent concentration (*C*_o_) occurs. The point of column exhaustion (*t*_e_) was determined when the effluent concentration reached 95% at a constant value of *C*_*t*_/*C*_o_ (Kundu et al. 2004).

*C*_*t*_/*C*_o_ as a function of time was used to elucidate the breakthrough curve. The total effluent volume (*V*_eff_ (mL)) passed through the column was calculated using Eq. (11). The total quantity of Pb(II) adsorbed (*q*_total_ (mg)) by the fixed-bed column was estimated using Eq. (12) from the area under the breakthrough curve (Chen et al. 2012). From the *q*_total_ value, the experimental maximum uptake capacity (*q*_bed_, mg g^−1^) of the column was calculated as shown in Eq. (13).

11 $$ {V}_{eff}=Q\times {t}_{total} $$

12 $$ {q}_{total}=\frac{Q}{1000}{\int}_{t=0}^{t= total}{C}_{ad} dt $$

13 $$ {q}_{bed}=\frac{q_{total}}{m} $$

The total amount of Pb(II) ions passed through the column system (*m*_total_) was calculated using Eq. (14). The removal percentage (RE, %) of Pb(II) can be calculated from Eq. (15). At equilibrium, the unadsorbed Pb(II) concentration (*C*_eq_) during the column process was obtained from Eq. (16):

14 $$ {m}_{total}=\frac{C_oQ{t}_{total}}{1000}\kern0.5em $$

15 $$ RE\ \left(\%\right)=\frac{q_{total}}{m_{total}}\times 100\kern0.5em $$

16 $$ {C}_{eq}=\kern0.5em \frac{m_{total}-{q}_{total}}{V_{eff}}\times 1000 $$

## Results and discussion

### Characterization studies

#### XRD analysis

**Fig. 1:**
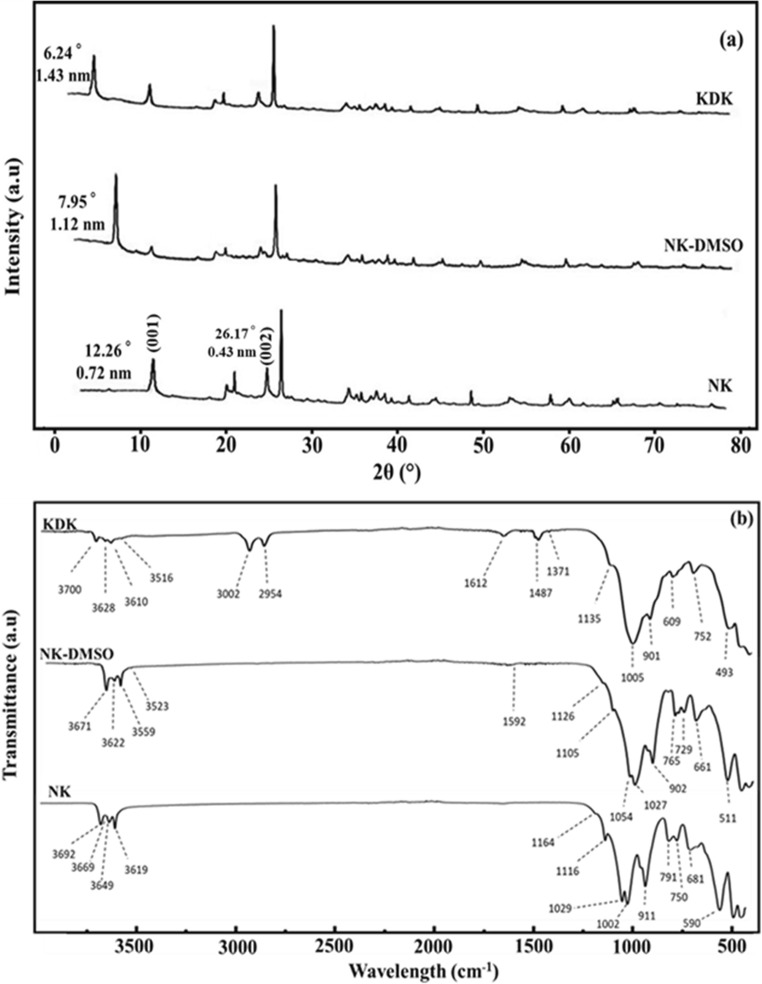

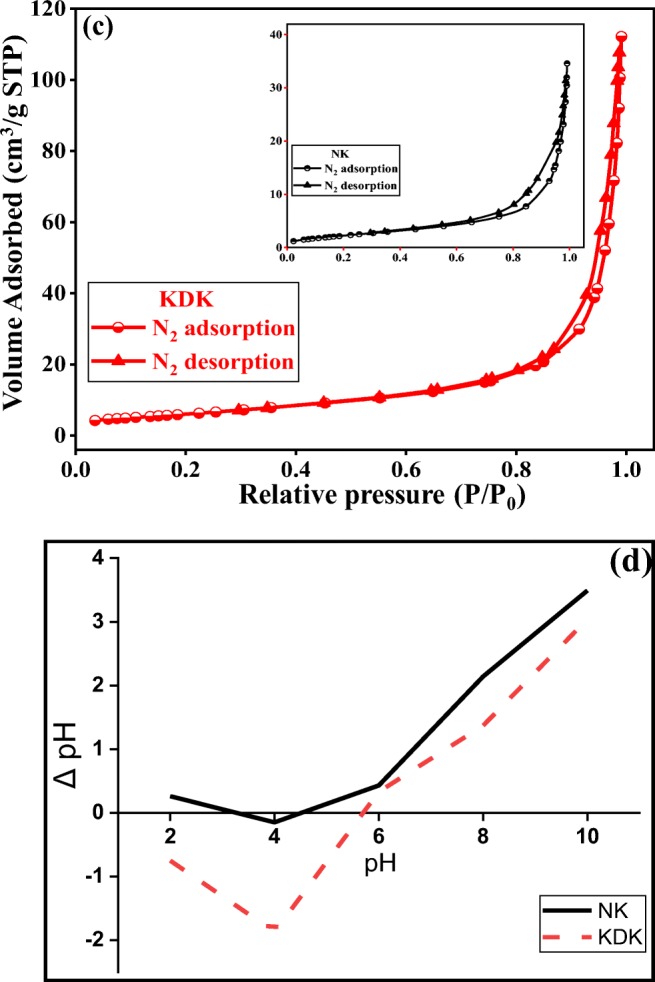
Sample characterization: **a** XRD patterns, **b** FT-IR spectra, **c** N_2_ adsorption–desorption isotherm, and **d** point of zero charge (pH_zpc_) measurements for NK and KDK

**Fig. 2:**
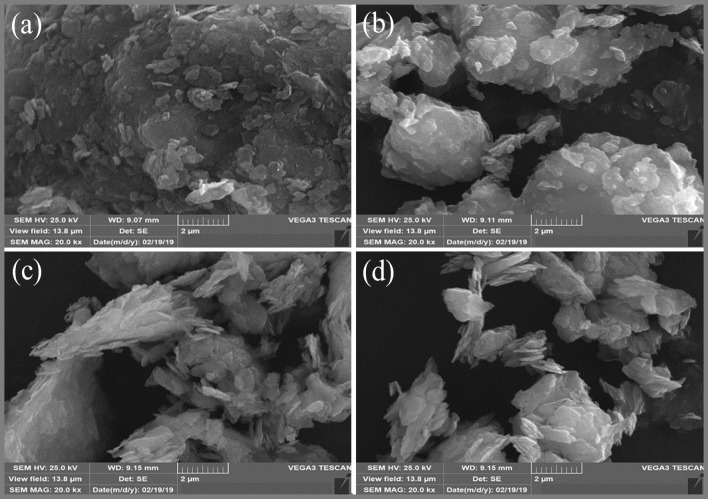
SEM images of intercalation stages: **a**, **b** NK, **c** NK-DMSO, and **d** KDK

The XRD patterns of NK, NK-DMSO, and KDK are illustrated in Fig. 1a. The characteristic XRD pattern of NK with reflections of the (001) and (002) planes was detected at *2θ* = 12.26° and 26.17°, respectively. Based on Bragg’s law, the interlayer spacing of kaolinite was calculated as *d*_(001)_ = 0.719 nm and *d*_(002)_ = 0.34 nm. After DMSO intercalation, the interlayer spacing of the raw kaolinite (0.719 nm) increased to 1.116 nm. This shift in *d*-value indicated that DMSO was successively intercalated into the kaolinite interlayers. The DMSO intercalation caused a structural expansion along the direction perpendicular to the (001) lattice plane (Δ*d* = 0.393). The loss of intensity and disappearance of some reflections in the high and middle 2*θ* angles could be attributed to the structural degradation of kaolinite after the intercalation reaction (Frost et al. 1998). The synthesized complex was intercalated again with K-Ac. After K-Ac intercalation (Fig. 2, KDK), the kaolinite (001) reflection was further shifted which was observed at 2*θ* = 6.24°. This shift caused further expansion of the interlayer spacing from 1.116 to 1.426 nm (Δ*d* = 0.707). The increase in interlayer spacing of the K-Ac intercalated complex could be attributed to the formation of hydrogen bonds through the coordinated water of Ac molecules and the oxygen atoms of the kaolinite layer (Deng et al. 2002). This hypothesis is based on the fact that acetate ions leached completely after immersing in water (Cheng et al. 2010a). Meanwhile, the K-Ac intercalation led to significant changes in the kaolinite’s surface properties (Table 1), which was confirmed with SEM analysis (Fig. 2d). Overall, the XRD pattern of KDK revealed large expansions of the kaolinite interlayers caused by DMSO and K-Ac intercalations. Therefore, the intercalation might improve the surface area and interlayer trapping ability for Pb(II) ions by the modified kaolinite.

**Table 1:** The textural properties of NK and KDK samples based on N_2_ adsorption–desorption isotherms

Properties	Samples	
	NK	KDK
BET surface area (m^2^ g^−1^)	8.473	24.87
*V*_m_ (cm^3^ (STP) g^−1^)	1.9467	5.0248
Total pore volume (cm^3^ g^−1^)	0.04956	0.165
Mean pore diameter (nm)	23.397	30.18
Langmuir surface area (m^2^ g^−1^)	6.4228	22.439
BJH surface area (m^2^ g^−1^)	10.398	26.341

#### FT-IR analysis

The NK, NK-DMSO, and KDK samples were characterized by FT-IR spectroscopy as shown in Fig. 1b. Kaolinite originally includes four different kinds of hydroxyl groups (outer-surface hydroxyl, inner-surface hydroxyl, inner hydroxyl, and absorbed water hydroxyl) (Kloprogge 2019) the presence of which was proven by absorption bands in the 3500–3800 cm^−1^ range. The four bands were observed (Fig. 1b, NK) at wavelengths 3692, 3669, 3649, and 3619 cm^−1^, respectively (Van der Marel and Beutelspacher 1976). The OH stretching region of the kaolinite inner surface might have a sensitive effect on the interlayer modification (Tunney and Detellier 1996). Thus, deformation of the FT-IR spectra in this range was commonly used to detect if the intercalating molecules would breakdown the original hydrogen bonds and establish new hydrogen bonds with the surface groups. Comparing the spectra in Fig. 1b, it was observed that in case of NK-DMSO, there was a disappearance of bands at 3669 and 3649 cm^−1^, and a decrease in the intensity and shifting of the band at 3619 cm^−1^. Meanwhile, new bands were observed at 3622, 3559, 3523, and 902 cm^−1^. The appearance of these new bands could be attributed to the hydrogen bonds between DMSO and kaolinite. Upon the intercalation of NK-DMSO with K-Ac (Fig. 1b, KDK), the disappearance of the bands at 3671, 3559, and 3523 cm^−1^ from NK-DMSO and the new appearance of bands at 3700 and 3628 cm^−1^ (which attributed to the hydroxyl group on the surface and internal structure of kaolinite) could be due to new hydrogen bond formation between kaolinite and K-Ac (Mehrotra and Giannelis 1991). In other words, the appearances of these bands in KDK indicated that the bonds between surface hydroxyls and DMSO molecules were removed from the surface of the kaolinite by K-Ac solution, subsequently establishing new bonds between the acetate and kaolinite surface (Mehrotra and Giannelis 1991). The bands at 3002 and 2954 cm^−1^ were attributed to the inner and outer plane vibration of the C–H bond from acetate, respectively. The band at 901 cm^−1^ was attributed to the hydroxyl deformation of the inner-surface hydroxyl groups that were hydrogen-bonded to the –S=O group of the DMSO molecule (Li et al. 2008). The appearance of band at 3610 cm^−1^ indicated that K-Ac molecules were intercalated into the clay mineral successfully. The appearance of new bands at 1612 and 1487 cm^−1^ could be attributed to the vibration bands of symmetric and antisymmetric stretching of CH_3_COO^−^ (Zhang et al. 2007), which also indicated that the CH_3_ group of acetate was interacting with the silica sheet (Cheng et al. 2010b).

#### N_2_ adsorption/desorption measurements

The N_2_ adsorption–desorption isotherms of NK and KDK are presented in Fig. 1c. The isotherms of NK and KDK were of IV type according to the IUPAC classification (Thommes et al. 2015). This isotherm type is characteristic of mesoporous materials (average pore diameter 2–50 nm) (Budsaereechai et al. 2012). From Fig. 1c, both curves for NK and KDK showed no significant difference. This indicated that the mesoporous character was preserved in the intercalated kaolinite. In addition, H3 hysteresis loops were observed for both NK and KDK, which indicated occupation and evacuation of the mesopores by capillary condensation (Thommes et al. 2015). Table 1 summarizes the surface properties using the standard BET method and Langmuir and BJH equations. The results revealed that the specific surface area of KDK was more than threefold higher than that of NK (Table 1). The obtained results illustrated that the thermal activation and intercalation processes significantly improved the surface properties of kaolinite. The total pore volumes were 0.05 and 0.17 cm^3^ g^−1^ for NK and KDK, respectively. These results also indicated that the intercalation method enhanced the kaolinite porosity. After kaolinite modification, the mean pore volume increased from 23.39 to 30.18 nm. This increase in the pore volume could explain the superior adsorption performance of Pb(II) ions by the modified kaolinite adsorbent (Table 1).

#### pH_zpc_ measurements

The pH_zpc_ determination helps to get insight into the adsorption process and in determining the surface charge of adsorbents. Based on the literature (Chintala et al. 2013; Divband Hafshejani et al. 2016), the adsorption is more favorable for cations when the solution pH is higher than pH_zpc_. On the contrary, the adsorption capacity is higher for anions when the solution pH is less than the pH_zpc_ (Parida et al. 1996). Therefore, the adsorbent surface is positively charged when the solution pH is less than pH_zpc_ and negatively charged above the pH_zpc_. As shown in Fig. 1d, the pH_zpc_ of NK and KDK was found to be 5.02 and 5.8, respectively. Thus, for the adsorption of Pb(II) cations by NK and KDK, a pH value higher than pH_zpc_ would be favorable.

#### SEM and EDX analysis

The change in the morphological features of kaolinite with different intercalation stages was evaluated through SEM analysis (Fig. 2). The SEM images of raw kaolinite showed that the kaolinite surface had a complex morphology (Fig. 2a, b) composed of small platelets arranged randomly varying in size from 0.2 to 2 μm. Additionally, the images showed the hexagonal edges and corners in some parts of the raw kaolinite structure. After the intercalation of NK-DMSO (Fig. 2c) and KDK (Fig. 2d), a significant morphological difference between the raw kaolinite and kaolinite intercalates was observed. After DMSO and K-Ac intercalation, the kaolinite grains changed to irregularly shaped large grains, which reflected the intercalation in the kaolinite layers. The comparative microscopic analysis of NK, NK-DMSO, and KDK showed that different intercalation processes provoked a severe modification of the kaolinite surface. It could be attributed to the lateral crystalline expansion during the thermal intercalation process that caused the breakage of the kaolinite interlayers (Du et al. 2010). Furthermore, it is important to consider that the changes in the surface morphology of the intercalates did not only depend on intercalation expansion, but also on external factors such as mechanical agitation, washing, grinding, and drying during the preparation steps. However, XRD and FT-IR results adequately supported the evidence of intercalation in the kaolinite structure.

**Fig. 3:**
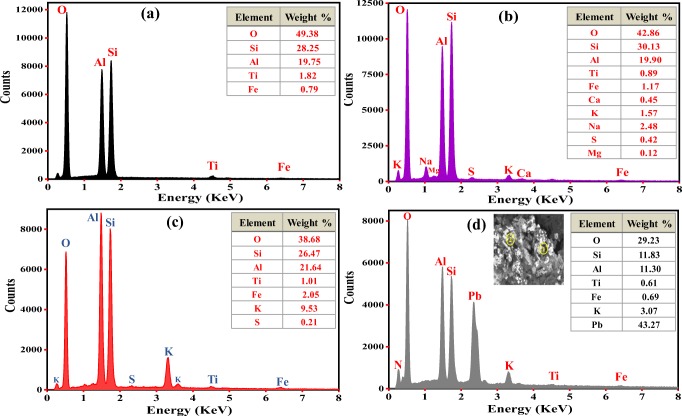
EDX spectra of **a** NK, **b** NK-DMSO, **c** KDK, and **d** KDK after Pb(II) adsorption

The chemical compositions on the surfaces of NK, NK-DMSO, and KDK were further examined by EDX analysis, and the results are shown in Fig. 3. The EDX analysis revealed that O, Si, and Al are the most abundant constituents in the samples. Additionally, the presence of Fe, K, Ti, Ca, Mg, and Na could be observed in the samples. The presence of these cations in the kaolinite samples was designated as polycationic kaolinite, which was favorable for the adsorption process (Maged et al. 2019). The results confirmed that there were no significant differences in the sample’s composition before and after intercalation except in S and K concentrations (Fig. 3b, c). The increase of S and K concentrations could be attributed to the compounds (DMSO and K-Ac) which were used in the intercalation process. Figure 3d shows the EDX analysis of KDK after Pb(II) adsorption. It was clearly observed that Pb(II) concentration appeared significantly after adsorption compared to KDK before adsorption (Fig. 3c), confirming that Pb(II) was successfully adsorbed onto KDK surfaces.

#### TEM analysis

**Fig. 4:**
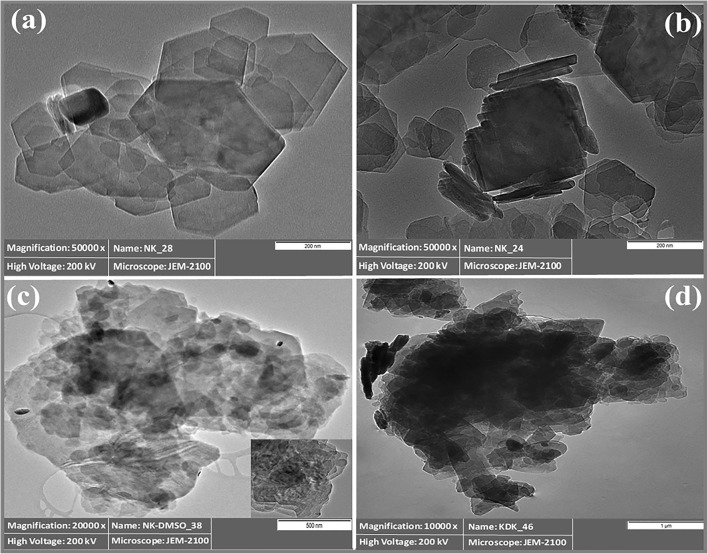
TEM images of intercalation stages: **a**, **b** NK, **c** NK-DMSO, and **d** KDK

TEM analysis was conducted in order to get an insight into the internal structural features of the kaolinite and its intercalated products. Figure 4 demonstrates the TEM images of raw kaolinite and the intercalates. Raw kaolinite grains exhibited well-defined hexagonal flakes, agreeing with the obtained results of the SEM images (Fig. 4a, b). After the DMSO intercalation, dark spots appeared between the kaolinite layers, which confirmed the kaolinite intercalation by DMSO and illustrated the increase of the *d*-value of NK-DMSO (Fig. 4c). After the K-Ac intercalation, a dark layer-like structure entirely filled the interlayer space of the kaolinite, which confirmed that the kaolinite layers were successfully intercalated (Fig. 4d). This phenomenon explained the expansion of kaolinite basal spacing (1.426 nm). Similar observations for kaolinite intercalation with other compounds (cetyltrimethylammonium bromide and sodium acetate) were reported by Shaban et al. (2018b).

### Kaolinite intercalation mechanisms

**Fig. 5:**
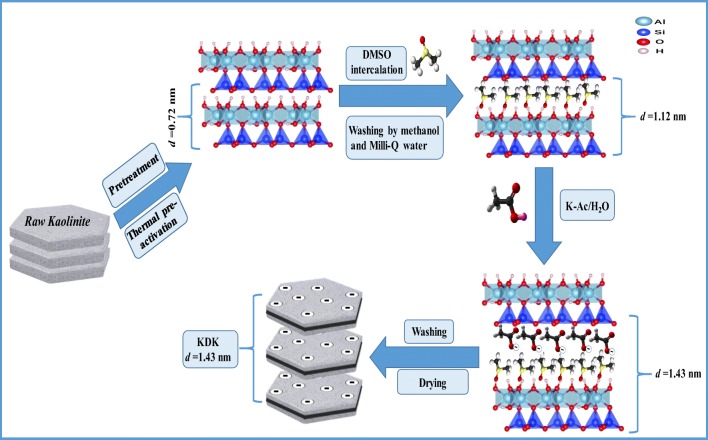
Schematic diagram for the mechanism of the intercalation stages of kaolinite

Figure 5 illustrates the mechanism of different stages of kaolinite intercalations. The kaolinite clay (1:1 type) is a layered silicate mineral. It originally consists of thick layers in nanometers formed by assembling an aluminum octahedron sheet and a silicon tetrahedron sheet along the direction (001) perpendicular to its crystal plane (Zhang et al. 2014). Kaolinite is a distinctively layered material having an asymmetric interlayer space that induces specific orientation of new guest molecules. In the first intercalation stage of kaolinite with DMSO, the DMSO molecules would weaken the cohesion energy between NK layers. This would promote the intercalation by forming hydrogen bonds between hydroxyl groups in the kaolinite surface and DMSO molecules. Thus, an increase of the interlayer space (Δ*d*) by 0.393 nm was confirmed with the XRD patterns (Fig. 5). In the second intercalation stage of NK-DMSO with K-Ac, the K-Ac was sandwiched in between kaolinite layers which promoted the intercalation due to the coordinated water molecules that were bound to the K-Ac molecules (Li et al. 2009). Hence, there was a further increase of the basal spacing (Δ*d*) by 0.707 nm which was confirmed by XRD analysis (Fig. 1a). The increase in the interlayer space (i.e., expanding interlayers) of the kaolinite thus provided a chance for increasing the Pb(II) adsorption sites.

### Batch adsorption experiments

#### Effect of initial pH on Pb(II) removal

**Fig. 6:**
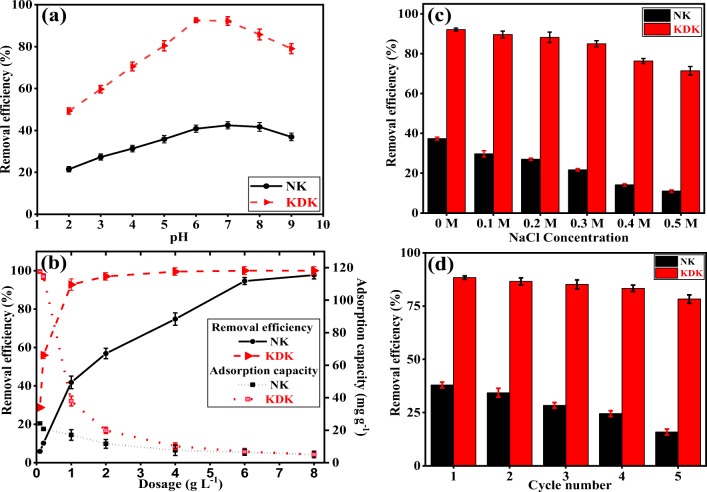
**a** Effect of pH, **b** effect of adsorbent dosage, and **c** effect of ionic strength (adsorbent dose 1 g L^−1^, Pb(II) initial concentration 40 mg L^−1^) for Pb(II) adsorption onto NK and KDK. **d** regeneration studies of Pb(II) adsorption onto NK and KDK using 0.1 M HCl as eluent

The initial pH of Pb(II) solution is one of the significant parameters that affects the adsorption of metal ions and the degree of ionization of functional groups on clay mineral surfaces. The effect of pH on the removal of Pb(II) ions by NK and KDK is illustrated in Fig. 6a. It shows that the removal efficiency was highly dependent on the solution pH values. Moreover, it was observed that an increase in the solution pH from 2 to 6 increased the removal efficiency. However, increasing the pH from 6 to 9 decreased the removal efficiency. This behavior could be explained by the presence of the negative charge on the adsorbent surface. The maximum amount of hydrogen ions (H^+^) at low pH would neutralize the negative charges on the adsorbent surface. Consequently, the presence of H^+^ ions reduced the available sites for Pb(II) adsorption. With an increase in the solution pH, a relative decrease in the amount of H^+^ would occur. In this case, the amount of adsorbed Pb(II) ions, which compete with H^+^, increased from pH 2 to 6. This behavior was confirmed by the pH_zpc_ measurements (Fig. 1d), which was found to be 5.02 and 5.8 for NK and KDK, respectively. Hence, the adsorbent surface was negatively charged above the pH_zpc_ and more favorable for Pb(II) cation adsorption. The Pb(II) precipitates and forms metal ion complexes (Pb(OH)^+^, $\mathrm{Pb}{\left(\mathrm{OH}\right)}_2^0$, and $\mathrm{Pb}{\left(\mathrm{OH}\right)}_3^{-}$) with the clay mineral surface at solution pH > pH 6 (Weng 2004). Thus, pH 6 was selected as the optimal pH for all further experiments.

#### Effect of adsorbent dosage

The effect of varying adsorbent dosages (0.1–8.0 g L^−1^) on the removal of Pb(II) is shown in Fig. 6b. The removal efficiency increased with an increase in adsorbent dosages from 6% (dose of 0.1 g L^−1^) to 98% (dose of 8.0 g L^−1^) for NK and from 29% (dose of 0.1 g L^−1^) to 100% (dose of 4.0 g L^−1^) for KDK. This behavior could be attributed to the increase in surface area and the number of binding sites with the increase in the mass of the adsorbent used, which favored the adsorption of pollutant. The Pb(II) adsorption was increased considerably when the adsorbent amount was higher than 1 g L^−1^. To compare the adsorption capacity of Pb(II) by the two adsorbents, the dosage of NK and KDK was selected as 1 g L^−1^.

#### Effect of ionic strength

The effect of electrolyte (NaCl) concentration in the aqueous solution has a significant role in Pb(II) adsorption by NK and KDK. Figure 6c shows that there was a decrease in the removal efficiency of NK and KDK for Pb(II) with increasing electrolyte concentrations from 0.1 to 0.5 M. Moreover, the removal efficiency of NK decreased from 37.41 (0 M NaCl) to 11.04% (0.5 M NaCl) and from 92.11 (0 M NaCl) to 71.46% (0.5 M NaCl) for KDK. This effect of ionic strength on Pb(II) adsorption could be due to the competition between Pb(II) ions and the electrolyte cations (Na^+^) for the binding sites. Thereafter, the electrolyte cations compete much more effectively with the negatively charged sites (Si–O) on kaolinite surface than on the aluminol sites (Al–OH) (Jiang et al. 2010). Therefore, a decrease in the adsorption efficiency with increasing electrolyte concentration would make the kaolinite surface less negatively charged, which would decrease the Pb(II) adsorption (Unuabonah et al. 2008).

#### Effect of contact time and adsorption kinetics

**Fig. 7:**
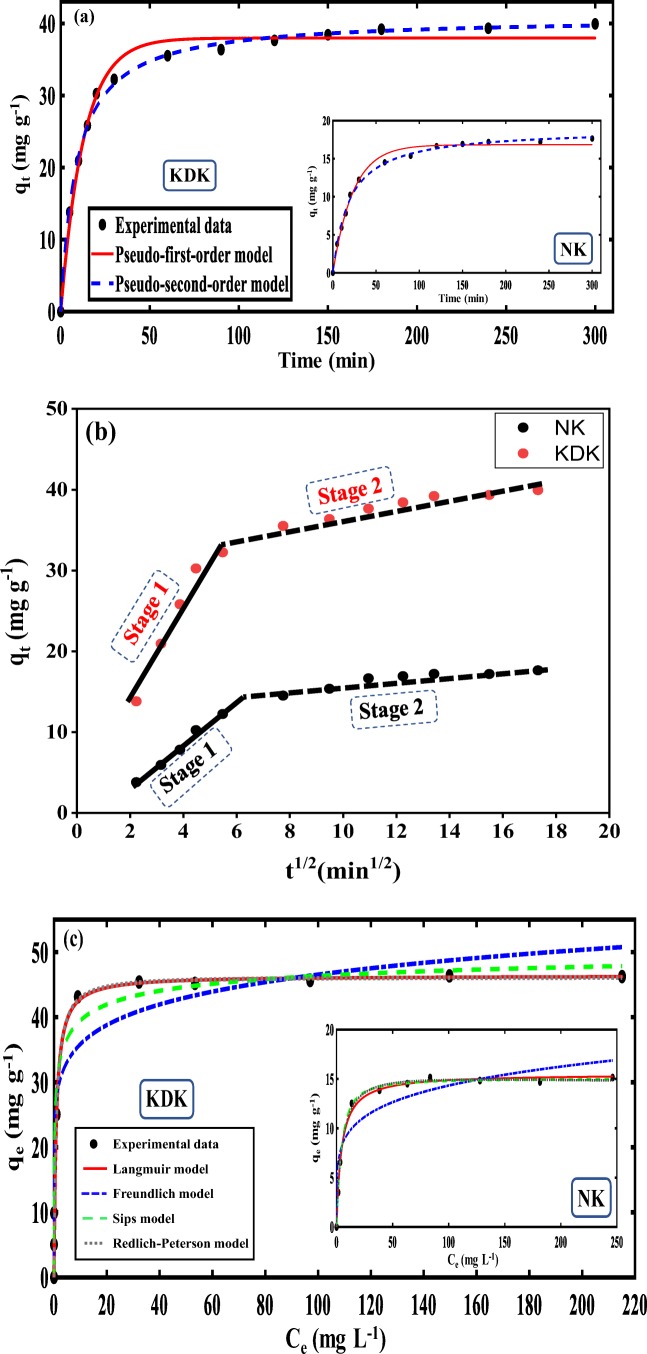
**a** PFO and PSO kinetics modeling, **b** intraparticle diffusion kinetics modeling, and **c** isotherm modeling for Pb(II) adsorption onto NK and KDK

Figure 4d shows the effect of contact time on the adsorption efficiency of Pb(II) onto NK and KDK. The kinetic studies were conducted for 5–300 min in the optimized experimental conditions (pH 6, dose 1.0 g L^−1^, initial concentration 40 mg L^−1^). As the experimental data shows (Fig. 7a), more than 90% of total adsorption occurred in the first 70 and 30 min for NK and KDK, respectively. The removal further increased with a much slower rate. Thus, 180 min was selected as the optimum contact time to investigate the adsorption kinetics of Pb(II) using NK and KDK.

The kinetic modeling of Pb(II) ions onto NK and KDK was assessed with the well-known kinetic models PFO and PSO models (Fig. 7a), and the resulting parameters are given in Table 2. The kinetic studies can give insight on the nature of the adsorption system (chemical or physical) and also the strength which is held between the adsorbate and adsorbent (Anbalagan et al. 2016; Abukhadra et al. 2019a, b). The experimental data showed that PSO fitted better than the results provided with PFO. The calculated adsorption capacity (*q*_e(cal)_) values and the experimental adsorption capacity (*q*_e(exp)_) values were compared in order to confirm the fitting results, which were close to each other. The correlation coefficient (*R*^2^) of the PSO was higher for NK and KDK than the obtained results of PFO (Table 2). This suggested that the chemisorption is important during the adsorption process, which involves cation exchange or chemical sharing process between Pb(II) ions and the adsorbents (Yin et al. 2018; Shaban et al. 2018a). Also, this model assumes that two different equilibrium phases occurred; the first one was fast which reached equilibrium quickly, whereas the second one was a slower reaction. Similar results were found by other researchers (Yin et al. 2018).

**Table 2:** Pseudo-first-order, pseudo-second-order, and intraparticle diffusion model parameters of Pb(II) onto NK and KDK

Adsorbent	Pseudo-first-order model				
	*q*_e(exp)_ (mg g^−1^)	*k*_1_ (min^−1^)	*q*_e(cal)_ (mg g^−1^)	RMSE	*R*^2^
NK	17.644	0.04285	16.83	0.5937	0.9907
KDK	39.94	0.07764	37.97	1.396	0.9866
Adsorbent	Pseudo-second-order model				
	*q*_e(exp)_ (mg g^−1^)	*k*_2_ (g mg^−1^ min^−1^)	*q*_e(cal)_ (mg g^−1^)	RMSE	*R*^2^
NK	17.644	0.002779	18.94	0.3954	0.996
KDK	39.94	0.002776	40.87	0.777	0.9962
Adsorbent	Intraparticle diffusion model				
	*q*_e(exp)_ (mg g^−1^)	*k*_id_ (mg g^−1^ min^−1/2^)	*C* (mg g^−1^)	RMSE	*R*^2^
NK	17.644	0.99033	3.89881	2.27306	0.916
KDK	39.94	1.86513	14.77	6.16637	0.846

##### Adsorption mechanisms

For the solid–liquid adsorption system, particle diffusion or film diffusion or both usually describe the solute transport phenomena (Anitha et al. 2015b). The adsorption of Pb(II) onto NK and KDK could be explained by the following four sequential steps: (1) the movement of Pb(II) ions from the aqueous solution to the external kaolinite surface (external or film diffusion), (2) the movement of Pb(II) ions from the external kaolinite surface into the internal part of the pores in the kaolinite layers (intraparticle or internal diffusion), (3) the Pb(II) ions get adsorbed into the interior surface of kaolinite (adsorption), and (4) chemical reaction via ion-exchange and/or complexation (the adsorption of Pb(II) ions into the active site on the kaolinite surface) (Rusmin et al. 2015). In order to understand the rate-controlling step and adsorption mechanisms, the IPD model was studied. The fitting of the IDP model is presented by plotting *q*_*t*_ versus *t*^1/2^ for NK and KDK (Fig. 7b). The IPD fitting curves for both the adsorbents showed two adsorption stages without intersection with the experimental data origin. The negative intersection of the IPD model indicated that intraparticle diffusion was not the only rate-limiting step. It also suggested that other mechanisms might be involved in the Pb(II) adsorption onto NK and KDK. Similar results were found by other authors previously (Rajkumar et al. 2013; Belhadri et al. 2019).

#### Effect of initial Pb(II) concentration and adsorption isotherm

In this study, the adsorption of Pb(II) ions onto NK and KDK was studied at the optimized experimental conditions. Figure 7c shows the adsorption capacity of the adsorbents at different initial Pb(II) concentrations between 5 and 250 mg L^−1^. The maximum adsorption capacity of Pb(II) onto NK and KDK was found to be 15.14 and 46.36 mg g^−1^, respectively.

Adsorption isotherms are mathematical models which clearly explain the relationship between the equilibrium concentrations of the adsorbate in the liquid phase and in the solid phase. The experimental isotherm data were fitted to four isotherm models (Langmuir, Freundlich, Sips, and Redlich–Peterson). As presented in Table 3 and Fig. 7c, the Sips, Redlich–Peterson, and Langmuir models were well fitted with the experimental data and provided a better fit than the Freundlich model for both NK and KDK. The *R*^2^ values were found to be 0.998 and 0.999 for NK and KDK, respectively, for the Sips model. The best fitting of the Sips model suggested that adsorption of Pb(II) took place both on heterogeneous and homogeneous surfaces of NK and KDK depending on the initial concentration of the pollutant (Sips 1948). Moreover, the theoretical maximum adsorption capacity (*q*_m_) for the Langmuir model was calculated to be 15.44 and 45.45 mg g^−1^ for NK and KDK, respectively. These values were close to the experimental adsorption capacity, which confirmed the applicability of the Langmuir model for describing the adsorption process. The Redlich–Peterson constant values (*g*) for NK and KDK were close to unity. This supported the fact that the adsorption of Pb(II) could be ideally described by the Langmuir model (Foo and Hameed 2010; Alexander et al. 2018).

**Table 3:** Langmuir, Freundlich, Sips, and Redlich–Peterson isotherm model parameters for Pb(II) adsorption onto NK and KDK

Adsorbent	Langmuir isotherm model					
	*q*_m_ (mg g^−1^)		*k*_L_ (dm^3^mg^-1^)	*R*^2^	RMSE	SSE
NK	15.52		0.2292	0.9953	0.4089	1.338
KDK	46.45		1.157	0.9994	0.4598	1.057
Adsorbent	Freundlich isotherm model					
	*K*_F_ (mg g^−1^) (L mg^−1^)^1/*n*^		*n*	*R*^2^	RMSE	SSE
NK	6.226		5.524	0.8954	1.921	29.53
KDK	30.23		10.28	0.9565	4.003	80.12
Adsorbent	Sips isotherm model					
	*K*_S_ (L g^−1^)	*β*_S_	*a*_S_	*R*^2^	RMSE	SSE
NK	15.06	1.269	0.1747	0.9985	0.2443	0.4177
KDK	53.15	1.152	1.152	0.9998	0.3281	0.4307
Adsorbent	Redlich–Peterson isotherm model					
	*K*_R_ (L g^−1^)	*a*_R_ (L/mg)	*g*	*R*^2^	RMSE	SSE
NK	3.138	0.17	1.036	0.9973	0.3296	0.7606
KDK	51.17	1.065	1.008	0.9996	0.4166	0.6941

#### Reusability of adsorbents

The reusability of the adsorbent is critical for the practical feasibility of adsorbents in water treatment. The reusability of NK and KDK was investigated by repeating the adsorption/desorption process for 5 cycles with a known volume of a 0.1 M HCl solution. As shown in Fig. 6d, NK and KDK exhibited good reusability with remarkable regeneration behavior. The adsorption percentage of Pb(II) onto NK was decreased significantly from 38 to 16% after 5 cycles. This could be attributed to the loss of some active adsorption sites during acid washing. However, the removal efficiency of Pb(II) onto KDK was slightly decreased from 89 to 78% after five consecutive adsorption–desorption cycles. These results suggested that KDK could be successfully regenerated by mild acid (HCl) treatment. The low-cost regeneration of KDK would facilitate the practical applications of the adsorbents in water treatment.

### Fixed-bed column adsorption studies

#### Effect of adsorbent loading

**Fig. 8:**
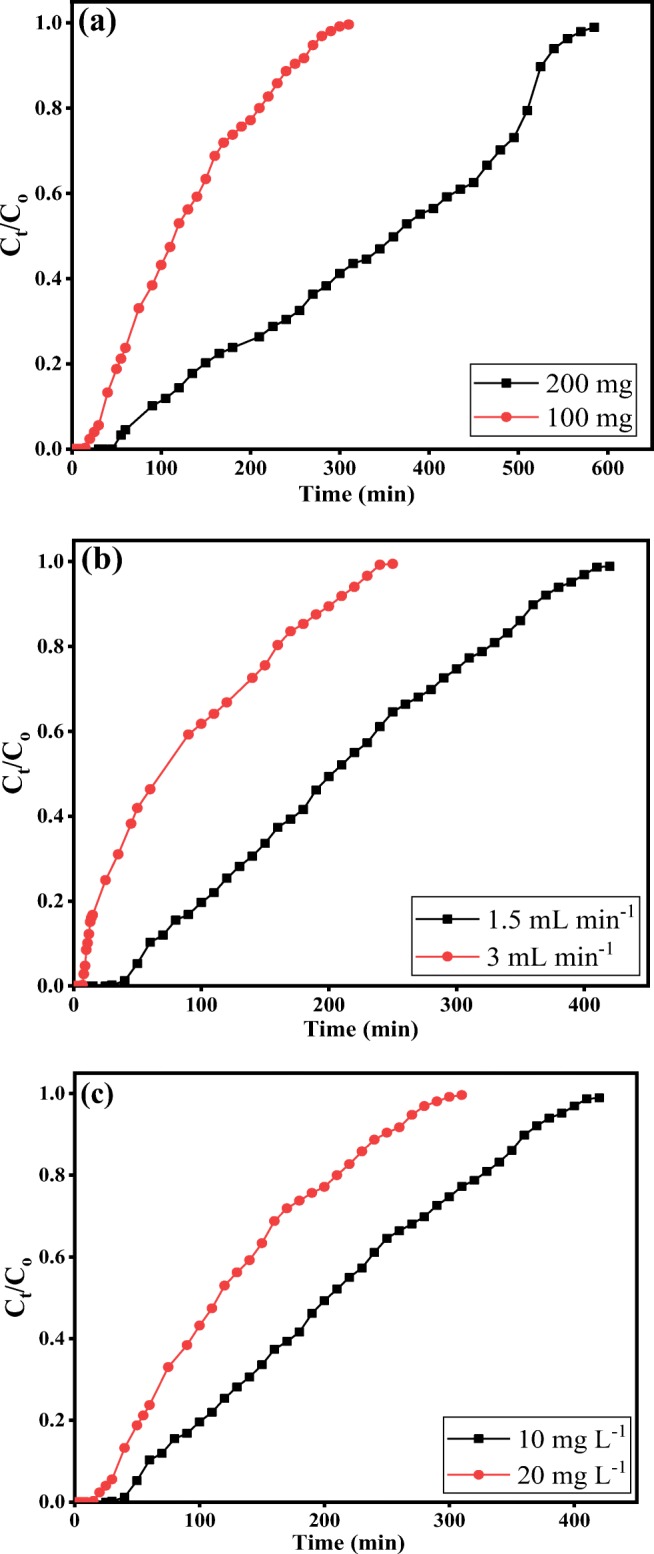
Effect of various parameters on the breakthrough curve of Pb(II) ions in the fixed-bed column onto KDK: **a** adsorbent loading 100 and 200 mg of KDK (conditions: initial Pb(II) 20 mg L^−1^, flow rate 1.5 mL min^−1^, pH 6), **(b)** flow rate 1.5 and 3 mL min^−1^ (conditions: initial Pb(II) 10 mg L^−1^, adsorbent loading 100 mg, pH 6), and **c** Pb(II) concentrations 10 and 20 mg L^−1^ (conditions: flow rate 1.5 mL min^−1^, adsorbent loading 100 mg, pH 6)

The effect of various adsorbent loadings on the breakthrough curve was studied using a fixed-bed column system. Based on the batch study results, the adsorbate solution (pH = 6) with initial Pb(II) concentration of 20 mg L^−1^ and flow rate 1.5 mL min^−1^ was passed through the column system with two different adsorbent loadings 100 and 200 mg. From Fig. 8a, it was observed that the steepness of the breakthrough curve was a function of adsorbent loadings in the column system. The breakthrough time was also found to decrease from 75 to 30 min with a decrease in the adsorbent loading from 200 to 100 mg. Table 4 represents the column parameters. The results exhibited that an increased adsorbent loading led to an increase in the effluent volume (*V*_eff_) from 465 (*m* = 100 mg) to 877.5 mL (*m* = 200 mg). Furthermore, the results provided in Table 4 exhibited an increase in the removal efficiency (RE%) for Pb(II) from 41.47 (*m* = 100 mg) to 57.95% (*m* = 200 mg). Similarly, the Pb(II) uptake in the column system (*q*_bed_) was found to be 52.55 and 39.86 mg g^−1^ when the adsorbent loadings were 100 and 200 mg, and the corresponding total Pb(II) adsorbed were 9.61 and 18.14 mg, respectively. The increase in total Pb(II) adsorbed with the increase of adsorbent loadings in the fixed-bed column could be attributed to the increased surface area of the adsorbent and increased number of available adsorption sites.

**Table 4:** The effect of flow rate, adsorbent loading, and initial lead concentration on the total adsorbed lead (*q*_total_), equilibrium uptake (*q*_bed_), total removal percentage (RE), and total unadsorbed lead concentration at equilibrium (*C*_eq_) for lead adsorption onto KDK

Experiments	Parameters									
	*C*_o_ (mg L^−1^)	*Q* (mL min^−1^)	*M* (mg)	*V*_eff_ (mL)	*t*_total_ (min)	*q*_total_ (mg)	*q*_bed_ (mg g^-1^)	*m*_total_ (mg)	RE (%)	*C*_eq_ (mg L^−1^)
Column 1	20.67	1.5	200	877.5	585	10.51	52.55	18.14	57.95	8.692
Column 2	20.67	1.5	100	465	310	3.99	39.86	9.61	41.47	12.09
Column 3	10.12	1.5	100	630	420	3.19	31.9	6.38	50.03	5.06
Column 4	11.09	3	100	750	250	2.96	29.55	8.32	35.53	7.15

#### Effect of flow rate

Figure 8b shows the effect of different flow rates on the breakthrough curve. The fixed-bed column adsorption experiments were carried out at two different flow rates (1.5 and 3 mL min^−1^). During the experiment, the influent concentrations, solution pH, and adsorbent loading were maintained as 10 mg L^−1^, 6 and 100 mg, respectively. Figure 8b illustrates the changes in breakthrough performance with different flow rates. Moreover, it was seen that the breakthrough time, exhaustion time, and adsorption efficiency were showing higher values at a lower flow rate (1.5 mL min^−1^). This behavior could be attributed to the fact that at the higher flow rate, the residence time of the adsorbate in the column system was becoming less. Thus, the interaction between the adsorbate and adsorbent was not enough to bind the metal ions to the adsorbent surface efficiently. In other words, if the residence time of the adsorbate in the column system was not large enough for adsorption equilibrium to be reached with a higher flow rate, the Pb(II) solution would leave the column system before equilibrium was achieved. This led to a lower removal efficiency of Pb(II) ions by KDK. Table 4 represents the experimental parameters calculated using the column data. It was evident that an increase in flow rate from 1.5 to 3 mL min^−1^ led to a decreased removal capacity (RE %) from 50.03 to 35.53% and simultaneously decreased Pb(II) uptakes in the column system (*q*_bed_) from 31.9 to 29.55 mg g^−1^, respectively. Furthermore, the concentration of unadsorbed Pb(II) increased in the effluent from 5.06 to 7.15 mg L^−1^ with an increase in flow rate from 1.5 and 3 mL min^−1^, respectively.

#### Effect of initial pollutant concentration

The column experiments were further conducted to investigate the effect of Pb(II) concentrations (10 and 20 mg L^−1^) on the breakthrough time. In this process, other parameters such as flow rate (1.5 mL min^−1^), solution pH (pH 6), and adsorbent loading (100 mg) were kept constant. Figure 8c exhibits that the breakthrough time reduced with an increasing Pb(II) concentration, and the exhaustion time was reached rapidly. This behavior could be attributed to the quick saturation of available active surface sites with an increase in influent Pb(II) concentration. Table 4 also reveals that increasing the influent concentration from 10 to 20 mg L^−1^ led to an increase in the equilibrium Pb(II) uptake (*q*_bed_) from 31.9 to 39.86 mg g^−1^ and an increase in the total Pb(II) adsorbed in the column system from 3.19 to 3.99 mg, respectively. However, it also showed that the removal efficiency decreased from 50.03 to 41.47% with an increasing influent concentration from 10 to 20 mg L^−1^, respectively.

### Comparison with other adsorbents

Table 5 shows a comparative study between the prepared adsorbents in this study (NK and KDK) and other adsorbents, as reported in the literature, for Pb(II) removal. Higher Langmuir capacity of intercalated kaolinite was observed as compared to other adsorbents. This could be due to the high surface area, enhanced interlayer space, unique porous properties, and presence of different types of functional groups on the modified kaolinite adsorbent. Thus, the intercalation of kaolinite was found as one of the best modification techniques, which could enhance its adsorption capacity for Pb(II) from aqueous solution. Some of the adsorbents shown in Table 5 displayed a higher adsorption capacity for Pb(II) than the results obtained in this work. It was possibly due to the difference in the modification method, structure, or specific surface area of adsorbents and different interactions between Pb(II) and the adsorbent.

**Table 5:** Comparison of maximum adsorption capacities of various raw and modified low-cost adsorbents reported in the literature for Pb(II) adsorption

Adsorbents	Adsorption capacity *q*_max_ (mg g^−1^)	
	Pb(II)	References
Natural palygorskite	104.28	Chen and Wang (2007)
Kaolinite nanotubes	89	Abukhadra et al. (2019a, b)
KDK	46.45	This study
Sewage sludge biochar	44.91	Ifthikar et al. (2017)
Acid activated bentonite	40.14	Eren et al. (2009)
Sodium polyphosphate/kaolinite	40.0	Amer et al. (2010)
Tree fern	40	Ho et al. (2004)
*Moringa olifera* bark	34.6	Reddy et al. (2010)
Meranti sawdust	34.25	Rafatullah et al. (2009)
Peanut husk	29.14	Li et al. (2007)
Pine cone–activated carbon	27.53	Momčilović et al. (2011)
Sawdust	21.05	Li et al. (2007)
Chitosan/PAN	20.08	Anitha et al. (2015a, b)
Egyptian kaolinite	15.52	This study
Kaolinite	7.75	Shahmohammadi-Kalalagh et al. (2011)
Chabazite	6.0	Ouki and Kavannagh (1997)

## Conclusions

This study exhibited that kaolinite was effectively modified by intercalation using DMSO and K-Ac for adsorbing Pb(II) ions from aqueous media. The XRD, FT-IR, SEM, and TEM characterization techniques confirmed that the kaolinite intercalation was successfully achieved. The improvement in Pb(II) adsorption occurred due to the increase in the kaolinite interlayer spacing, which increased the available adsorption sites on the adsorbent. The adsorption of Pb(II) by KDK was best explained by the Sips model. The maximum uptake capacity was found to be 15.14 and 46.36 mg g^−1^ for NK and KDK, respectively. The adsorption kinetics was rapid and more than 90% of the total adsorption was reached within 70 and 30 min for NK and KDK, respectively. The PSO kinetic model was the best model for describing the Pb(II) adsorption. The Pb(II) adsorption by the clay adsorbent was found to be influenced by initial pH and electrolyte concentration in the aqueous solution. The KDK performed well under environmental pH and ionic strength conditions. Regeneration studies showed that KDK could be reused for 5 cycles using 0.1 M HCl as an eluent. The maximum adsorption capacity of Pb(II) in a fixed-bed column for KDK was found to be 52.55 mg g^−1^. Moreover, it was observed that the removal percentage of Pb(II) increased with an increase in the adsorbent loading, while it decreased with an increase in both influent concentration and flow rate. The intercalated kaolinite has the potential to be used as an adsorbent for the removal of Pb(II) ions from aqueous media. The intercalated kaolinite adsorbent reported in this paper may also be helpful to remediate other co-occurring contaminants such as nickel, zinc, cobalt, copper, cationic pharmaceuticals, and dyes in contaminated aqueous media.

## Electronic supplementary material

ESM 1 (DOCX 20 kb)

## Nomenclature

*θ*: The angle of diffraction (°)
*d*: The basal spacing (°A)
*n*_B_: The path differences between the reflected waves which equals an integral number of wavelengths (*λ*)
*q*_e_: The adsorption capacity of the adsorbent (mg g^−1^)
*C*_i_: The initial Pb(II) concentration (mg L^−1^)
*C*_e_: The equilibrium Pb(II) concentration (mg L^−1^)
W: The mass of clay adsorbent (g)
*V*: The volume of Pb(II) solution (L)
*q*_*t*_: The adsorption capacity (mg g^−1^) at time *t* (min)
*k*_1_: The constants of PFO (min^−1^)
*k*_2_: The constants of PSO (g mg^−1^ min^−1^)
*k*_id_: The constants of IPD (mg g^−1^ min^−1/2^)
*q*_m_: The Langmuir constant associated with adsorption capacity (mg g^−1^)
*K*_L_: The constants of Langmuir (L mg^−1^)
*K*_F_: The constants of Freundlich (mg g^−1^) (L mg^−1^)^1/*n*^
*n*: The exponent of Freundlich related to adsorption intensity
*K*_s_: The Sips constant (L mg^−1^)
*K*_R_: The Redlich–Peterson constant (L g^−1^)
*α*_R_: The Redlich–Peterson constant (mg^−1^)
*g*: The Redlich–Peterson exponent
*R*^2^: The correlation coefficient
RMSE: The root mean square error
*Q*: The flow rate (mL min^−1^)
*t*_total_: The total flow time (min)
*C*_ad_: The adsorbed Pb(II) concentration (mg L^−1^)
*m*: The KDK dry mass (g)
XRD: X-ray diffraction
FT-IR: Fourier transform infrared
SEM: Scanning electron microscope
EDX: Energy-dispersive X-ray
TEM: Transmission electron microscope
BET: Brunauer–Emmett–Teller
BJH: Barrett–Joyner–Halenda
IUPAC: The International Union of Pure and Applied Chemistry
