# Supplementary material for: Enhanced interlayer trapping of Pb(II) ions within kaolinite layers: intercalation, characterization, and sorption studies
Source: Environ Sci Pollut Res Int. 2019 Nov 23;27(2):1870–87. doi: 10.1007/s11356-019-06845-w (PMC6994523; doi:10.1007/s11356-019-06845-w)
Supplement: Supplementary file 1 — (DOCX 20 kb) [file 11356_2019_6845_MOESM1_ESM.docx]

**Enhanced interlayer trapping of Pb(II) ions within kaolinite layers: intercalation, characterization and sorption studies**

Ali Maged^1,2,*^, Ismael Sayed Ismael^2^, Sherif Kharbish^2^, Binoy Sarkar^3^, Sirpa Peräniemi^4^,

Amit Bhatnagar^1^

*^1^ Department of Environmental and Biological Sciences, University of Eastern Finland, P.O. Box 1627, FI-70211 Kuopio, Finland*

^2^ *Geology Department, Faculty of Science, Suez University, El Salam City, P.O. Box 43518, Suez Governorate, Egypt*

^3^*Department of Animal and Plant Sciences, The University of Sheffield, Western Bank, Sheffield, S10 2TN, UK*

^4^ *School of Pharmacy, University of Eastern Finland, FI-70211, Kuopio, Finland*

* Corresponding author.

Email addresses: [Ali.Maged@suezuni.edu.eg](mailto:Ali.Maged@suezuni.edu.eg) (A. Maged)

*S1. Physicochemical analysis*

The X-ray diffraction (XRD) analysis of NK, NK-DMSO and KDK samples was conducted using a Philips powder-diffractometer (model PW1050, Ni-filtered Cu-K_α_ radiation, USA). The accelerating voltage was set as 40 kV, and the current was 30 mA. The scanning was limited from 3 to 80° on the *2θ* angle. In order to determine the interlayer space of kaolinite, the *d*-values was calculated according to Bragg´s law (Bragg and Bragg 1913) using the following Eq. (S1).

$n_{B}\lambda=2d\sin\theta$ (S1)

Fourier transform infrared (FT-IR) analysis of NK, NK-DMSO, and KDK was performed using a Bruker ALPHA ATR-FTIR spectrometer equipped with a diamond crystal and a Rock-Solid interferometer. The spectra were obtained within the range of 4000–400 cm^-1^ with 256 scans per sample at 4 cm^-1^ resolution. The N_2_ adsorption/desorption measurements for the evaluation of the specific surface area (Brunauer-Emmett-Teller (BET), Langmuir and Barrett-Joyner-Halenda (BJH)) were performed at -196 °C using a Belsorp Mini II system (Japan).

The surface morphology of the raw kaolinite and the modified samples at different intercalation stages (NK, NK-DMSO, and KDK) was analyzed using a scanning electron microscope (SEM) (Tescan VEGA3 electron microscope) at different magnifications ranging from 5 to 30 kx. Energy-dispersive X-ray (EDX) analysis was conducted for investigating the chemical composition of samples and to determine the alteration in chemical composition before and after the intercalation (EDX, Thermo Pathfinder v1.4, Madison, WI, USA). The acceleration voltage of 15 kV and nitrogen gas pressure of 20 Pa in the vacuum chamber was used for discharging the electron exposure off the nonconducting specimen surface during low vacuum operation. A transmission electron microscope (TEM) (JEM-2100F, JEOL Co., Tokyo, Japan) was used to get further details about the internal morphological and structural features of the samples. Images were acquired using 200 kV field emission gun.

The point of zero charge *(pH_zpc_)* measurements of NK and KDK were conducted by dispersing 0.05 g of the adsorbent in 25 mL of 10 mM NaCl aqueous solution. HCl (0.1 M) and NaOH (0.1 M) were used (before adding the adsorbent) to adjust the solution initial pH. The samples were shaken at 200 rpm in a multi-functional orbital shaker (Grant-bio, UK) at constant temperature 25 ± 1 °C for 24 h in glass bottles. Afterward, the suspension pH values at the equilibrium were measured. The deviation in the solution pH (ΔpH) versus the initial pH (pH_i_) was plotted for both adsorbents. Thereafter, the pH_zpc_ value was found from the intersection point of the resulting plotted curves (Shirani et al. 2018).

**References**

Bragg WH, Bragg WL (1913) The Reflection of X-rays by Crystals. Proc R Soc A Math Phys Eng Sci 88:428–438. doi: 10.1098/rspa.1913.0040

Shirani Z, Santhosh C, Iqbal J, Bhatnagar A (2018) Waste Moringa oleifera seed pods as green sorbent for efficient removal of toxic aquatic pollutants. J Environ Manage 227:95–106. doi: 10.1016/J.JENVMAN.2018.08.077
